# Encouraging Brand Evangelism Through Failure Attribution and Recovery Justice: The Moderating Role of Emotional Attachment

**DOI:** 10.3389/fpsyg.2022.877446

**Published:** 2022-05-19

**Authors:** Tingting Zhu, Sung Kyu Park

**Affiliations:** ^1^School of Business, Research Institute of Decision and Behavior Science, Anhui University of Technology, Ma’anshan, China; ^2^Department of International Trade, Changwon National University, Changwon, South Korea

**Keywords:** failure attribution, recovery satisfaction, brand evangelism, emotional attachment, recovery justice

## Abstract

Brand evangelism is essential to the profitability of e-shops, but the effects of failure attribution and recovery justice in encouraging brand evangelism in the online service recovery context are not straightforward. Grounded on a framework integrating Attribution theory, Justice theory, and Attachment theory, this study explores whether failure attribution and recovery justice affect brand evangelism through recovery satisfaction with emotional attachment as a moderator. We gathered 400 samples from e-shoppers who encountered a service failure and recovery in the past year to verify the hypotheses using structural equation modeling and multiple-group analysis. Results declare that failure attribution (locus, stability, and controllability) and recovery justice (distributive, procedural, and interactional justice) are significantly related to recovery satisfaction and subsequent brand evangelism. Moreover, failure attribution (locus, stability, and controllability) correlates significantly with recovery justice (distributive, procedural, and interactional justice). In addition, emotional attachment plays a moderating role on the relationships between distributive and procedural justice on recovery satisfaction. This work contributes to brand evangelism research by giving a different perspective (i.e., service recovery) to comprehend what stimulate or deter brand evangelism. In addition, this work develops service recovery research through the combination of the third dimension of attribution (locus) and fourth dimension of justice (informational justice) into a framework, investigating the effect of failure attribution on recovery justice, and revealing the moderating effect of emotional attachment in the recovery process.

## Introduction

In a highly competitive service industry, even a tiny mistake in the service delivery process can increase customer switching intention (Wei and Lin, 2020). Due to the unique features of service, service failure is unavoidable (Manu and Sreejesh, 2021; Shamim et al., 2021). Immediate action to perform well-executed recovery can help restore customer satisfaction (Cantor and Li, 2018; Mazhar et al., 2022). Service recovery is a critical factor in gaining customer satisfaction and positive behavioral intentions (Kima and Baker, 2020; Zhu et al., 2021).

Prior studies use attribution theory to interpret consumer reactions to service failure (Belanche et al., 2020). Attribution is an attempt to explain why a specific failure occurs (Van Vaerenbergh et al., 2014). Attribution theory, stemming from social psychology, is regarded as a critical construct in marketing. It is vital to understand the attribution process both theoretically and practically, because customers are likely to attribute negative outcomes (Xu et al., 2020). Generally, prior studies have only taken into account of two attribution dimensions (stability and controllability; Kim and Cho, 2014). To date, only a handful of studies have looked at the impact of attribution by including locus dimension (Harrisa et al., 2006; Oflac et al., 2021). In fact, the three-dimensional model is superior to the two-dimensional model (Moliner-Velázquez et al., 2015). So it could be interesting to include locus as an independent variable in service failure and recovery research.

Scholars have employed justice theory as an important research frame for analyzing service recovery strategies (McColl-Kennedy and Sparks, 2003). The logical basis of the theory is that customers’ perception of recovery justice has a great impact on their mentality and behavior (Akram et al., 2019, 2021; Khan et al., 2021). So it is vital for service providers to have a clear understanding of justice dimensions to work out effective recovery strategies. A large amount of service recovery research just thinks about three justice dimensions (distributive, procedural, and interpersonal justice; Tax et al., 1998; Smith et al., 1999; Chebat and Slusarczyk, 2005; Collier and Bienstock, 2006). Limited research has investigated the influence of recovery justice by including informational justice dimension (Bhatti and Khattak, 2015; Nikbin et al., 2015; Ngahu et al., 2016). Colquitt (2001) stated that the four-dimensional model outstrips the three-dimensional model (Colquitt, 2001). Thus, it is necessary to include informational justice as the fourth justice dimension in the service recovery study.

Scholars have analyzed service recovery from the perspective of failure attribution and recovery justice. However, little is known about the impact of failure attribution on recovery justice. Only few studies have empirically analyzed the influence of customers’ failure attribution on their recovery justice (Burton et al., 2014; Schneider and Castillo, 2015). Therefore, we proposed a research model integrating three attribution dimensions (locus, stability, and controllability) with four justice dimensions (distributive justice, procedural justice, interactional justice, and informational justice), and empirically investigate the correlation between failure attribution and recovery justice to fill in the research gap.

Service marketing scholars have pointed out that relationship marketing should be developed to a higher level, not limited to customer loyalty (Fierro et al., 2014; Gohary et al., 2016b; Odoom et al., 2020). Brand evangelism, an amplification of word-of-mouth intention (Nyadzayo et al., 2020), is worth considering as an important concept (Doss, 2014). It should be investigated in the context of service recovery, because understanding how to incentivize customers’ evangelism of the brand in service recovery allows firms to turn a crisis into an opportunity. But empirical studies have rarely been conducted, leading to a pressing need for brand evangelism research in the service recovery setting (Abd Rashid et al., 2017). Besides, according to previous studies, failure attribution and recovery justice affect customers’ behavioral intentions, such as word-of-mouth and patronage intention (Blodgett et al., 1997; Kim et al., 2009; Harrison-Walker, 2019). But the roles of failure attribution and recovery justice in predicting brand evangelism have not been verified. This study intends to advance our knowledge of brand evangelism literature by exploring the relationships of failure attribution and recovery justice to recovery satisfaction and subsequently to brand evangelism in the context of online service recovery.

Furthermore, the moderating role of emotional attachment, which is very important but relatively under-emphasized in the service recovery literature, was discussed in this study. Customer behaviors are mostly emotion-oriented, and in particular, emotion occupies a vital position in customer attitude and behavior (Río-Lanza et al., 2009; Wen and Chi, 2013; Kima and Baker, 2020; Babin et al., 2021). Emotional attachment is described as the emotional bond that connects a person with a specific purpose (Jimenez and Voss, 2014). It is an essential concept in the marketing literature. Prior studies have supported the direct effect of emotional attachment on recovery satisfaction (Wen and Chi, 2013; Nguyen and Minh, 2018), as well as the mediating role of emotional attachment between service recovery activities and recovery satisfaction (Río-Lanza et al., 2009; Vázquez-Casielles et al., 2012). However, few studies have delved into its moderating role in service recovery (Esen and Sonmezler, 2017; Torres et al., 2020). Joireman et al. (2013) suggested that future research should study how emotion influences customers’ assessment of service recovery (Joireman et al., 2013). Hence, this study focused to examine the moderating role of emotional attachment in the context of recovery justice and recovery satisfaction to gain a better understanding of customers’ assessment process of service recovery.

The main research purpose of this paper is to narrow gaps in literature through exploring how failure attribution and recovery justice encourage brand evangelism in the online service recovery context. More specifically, this study aims to: (1) verify whether failure attribution is related to recovery justice; (2) investigate whether failure attribution and recovery justice influence recovery satisfaction and subsequent brand evangelism; and (3) examine whether emotional attachment has a moderating effect in the relationship between recovery justice and recovery satisfaction.

This study contributes to service recovery and brand evangelism literature in three ways. First, different from prior research, this study includes the third dimension of attribution (locus) and fourth dimension of justice (informational justice) as independent variables. In particularly, this study ascertains the influence of failure attribution on recovery justice. Second, this study extends brand evangelism to service recovery context by probing into the mechanism through which failure attribution and recovery justice affect brand evangelism. Third, this study sheds light on recovery justice-recovery satisfaction mechanism by examining the moderating effect of emotional attachment, which provides a useful supplement for previous literature underestimating the moderating effect of emotional attachment. Therefore, this study has developed a more comprehensive framework than prior research to comprehend how to encourage brand evangelism from online service recovery.

## Literature Review

### Failure Attribution

Attribution theory originated from social psychology theory. It has become a hot research topic in various fields. Researchers pointed out that when a service failure occurs, customers tend to search for the cause of the failure (Folkes, 1984; Bitner et al., 1990; Zhu et al., 2013). Failure attribution is defined as the actual cause of the service failure inferred by dissatisfied consumers (Lee et al., 2020). It has a vital impact on customer behavior (Sukariyah and Assaad, 2015). Failure attribution is often classified into two dimensions: controllability (that is whether the service provider could have prevented service failure) and stability (that is whether the cause of service failure is constant; Weiner, 1979). However, locus (that is who should take responsibility for the failure) also affect recovery expectations (Harrisa et al., 2006) and the justice perceptions in recovery (Oflac et al., 2021). Thus, this study suggests that failure attribution should comprise the following dimensions.

First, locus is whether the service failure is attributable to themselves or the firm (Hess et al., 2003). When dissatisfied customers attribute the failure to themselves, in other words, due to the internal attribution, the service provider takes low responsibility for the loss, and the converse is also true. There are cases that customers blame themselves for the negative experience (Bowling and Michel, 2011). In addition, customers who regard the causes of service failure are because of internal reasons consider the behavior of the service providers to be proper (Bowling and Beehr, 2006). According to previous studies, when consumers think external reasons as causes of service failure, they tend to have negative feelings (Oflac et al., 2021). This study focuses on the service failure that customers attribute to the service provider for the cause of service failure.

Second, stability indicates whether the customer considers the cause of service failure as temporary or constant (Folkes, 1984). Customer will be more dissatisfied if the cause of a potential service failure is more likely to be due to a stable cause rather than an unstable cause (Hess et al., 2003). In other words, if a service failure occurs due to a stable cause, the customer will perceive a sense of injustice, thereby exacerbating customer dissatisfaction. This study focuses on the service failure that customers perceive it to be due to a stable cause.

Third, controllability means whether the cause of failure is within the control of the firm. That is, the degree to which customer perceives the cause as intentional or unintentional (Hess et al., 2003). When customers attribute unsatisfactory service failure experiences to causes that are beyond the service provider’s control, they do not think failure is because of intentional behavior (Weiner, 2000). On the contrary, if customers deem that service failure is preventable, they tend to perceive it as an unfair occurrence, which will lead to a dissatisfaction with the service provider. This study focuses on the service failure that customers perceive it as controllable regarding the cause of service failure.

### Recovery Justice

Based on social psychology literature and organizational psychology literature, justice theory takes an important place in the theoretical framework of service recovery literature (Wirtz and Mattila, 2004). This study uses justice theory to understand customers’ reactions to recovery efforts. Justice theory claims that the fairness of recovery strategy provided by service providers depends on customer’s feelings (McColl-Kennedy and Sparks, 2003). Considering that customers evaluate recovery efforts according to the perception of justice, the study on recovery justice is essential.

In general, justice dimensions include distributive, procedural and interactional justice (Blodgett et al., 1997; Tax et al., 1998; Smith et al., 1999; Chebat and Slusarczyk, 2005). But Colquitt (2001) declared that the four-dimensional model which includes informational justice surpasses the three-dimensional model, because the veracity of information plays an important role in service recovery process (Colquitt, 2001). Therefore, this study proposes that recovery justice should be four dimensions, which are connected with financial rewards (distributive justice), systems and policies (procedural justice), complaint settlement efforts (interactional justice), explanations for service failure and recovery (informational justice), respectively.

Scholars reveal that recovery satisfaction and behavioral intention after service recovery vary in accordance with the degree of recovery justice (Lii et al., 2012; Mostafa et al., 2015; Musiiwa et al., 2020; Roy et al., 2022). Service providers should analyze the impacts of justice dimensions on customers’ assessment process of service recovery to make successful recovery strategies (Gohary et al., 2016a).

### Recovery Satisfaction

Customer satisfaction has been a popular topic of marketing and consumer behavior research (Wei et al., 2021). No company can overlook the significance of delivering the best service to gain customer satisfaction (Rashid and Ahmad, 2014). In general, customer satisfaction is a pleasant state of consumers when their needs and desires are met (Oliver, 1997).

Recovery satisfaction is vital for the firm (Zhu et al., 2020), because if customers are dissatisfied with recovery efforts, they tend to exhibit negative attitudes (Alenazi, 2021). Recovery satisfaction is defined as customers’ positive emotions resulting from problem-solving performed by service providers (Kim et al., 2009).

### Brand Evangelism

As a stretching of word-of-mouth marketing, brand evangelism is not limited to the behavior of sharing positive words about a specific product or service with other customers, but actively influencing others to consume the same brand and dissuading others from using the competitor’s brand (Doss, 2014). It includes behaviors of the will to protect the brand from negative word-of-mouth, and become an informal spokesperson for a product or service (Becerra and Badrinarayanan, 2013). Word-of-mouth means informally providing consumers with opinions on brand evaluation, while brand evangelism is a method of delivering opinions to consumers with credibility and expertise. The study of brand evangelism is still in its infancy and little attention has been given to encouraging brand evangelism in service recovery (Rashid and Ahmad, 2014). Because brand evangelism is firmly rooted in word-of-mouth, and many service recovery studies reveal recovery satisfaction as an important antecedent of positive word-of-mouth (Maxham and Netemeyer, 2002; Wen and Chi, 2013), this study examines how to encourage brand evangelism through recovery satisfaction from the perspective of failure attribution and recovery justice.

### Emotional Attachment

In the marketing literature, emotional attachment is an important relationship-based concept that refers to the emotional bond with a consumption entity, such as brand, person, place or commodity (Park and Macinnis, 2006). This bond influences customer behavior and increases the profitability and productivity of the firm (Thomson et al., 2005).

The construct of emotional attachment was derived from the attachment theory of psychology started by Bowlby (1980). Attachment is an affectional tie with parents acquired by people from babyhood. Later in their lives, people develop attachments to objects (Leets et al., 1995). Marketing studies have demonstrated that attachment can transcend ownership, place, and individual relationships to a store or brand (Kleine and Baker, 2004; Carroll and Ahuvia, 2006). When a consumer purchases a brand reflecting his/her personality, a good experience leads to a positive brand attitude, contributing to brand attachment.

Researchers found that customers can develop an emotional attachment to various objects, such as gifts, brands (Patwardhan and Balasubramanian, 2011). In the past decade, emotional attachment to the brand has got more and more attention in the marketing literature. Extant studies have examined the influence of emotional attachment on recovery satisfaction as an antecedent (Kim et al., 2009; Wen and Chi, 2013; Nguyen and Minh, 2018) or a mediator (Río-Lanza et al., 2009; Vázquez-Casielles et al., 2012). But little attention has been paid to its role as a moderator (Esen and Sonmezler, 2017; Torres et al., 2020), which leads to a need for more work.

## Conceptual Model and Hypotheses Development

### Conceptual Model

**Figure 1:**
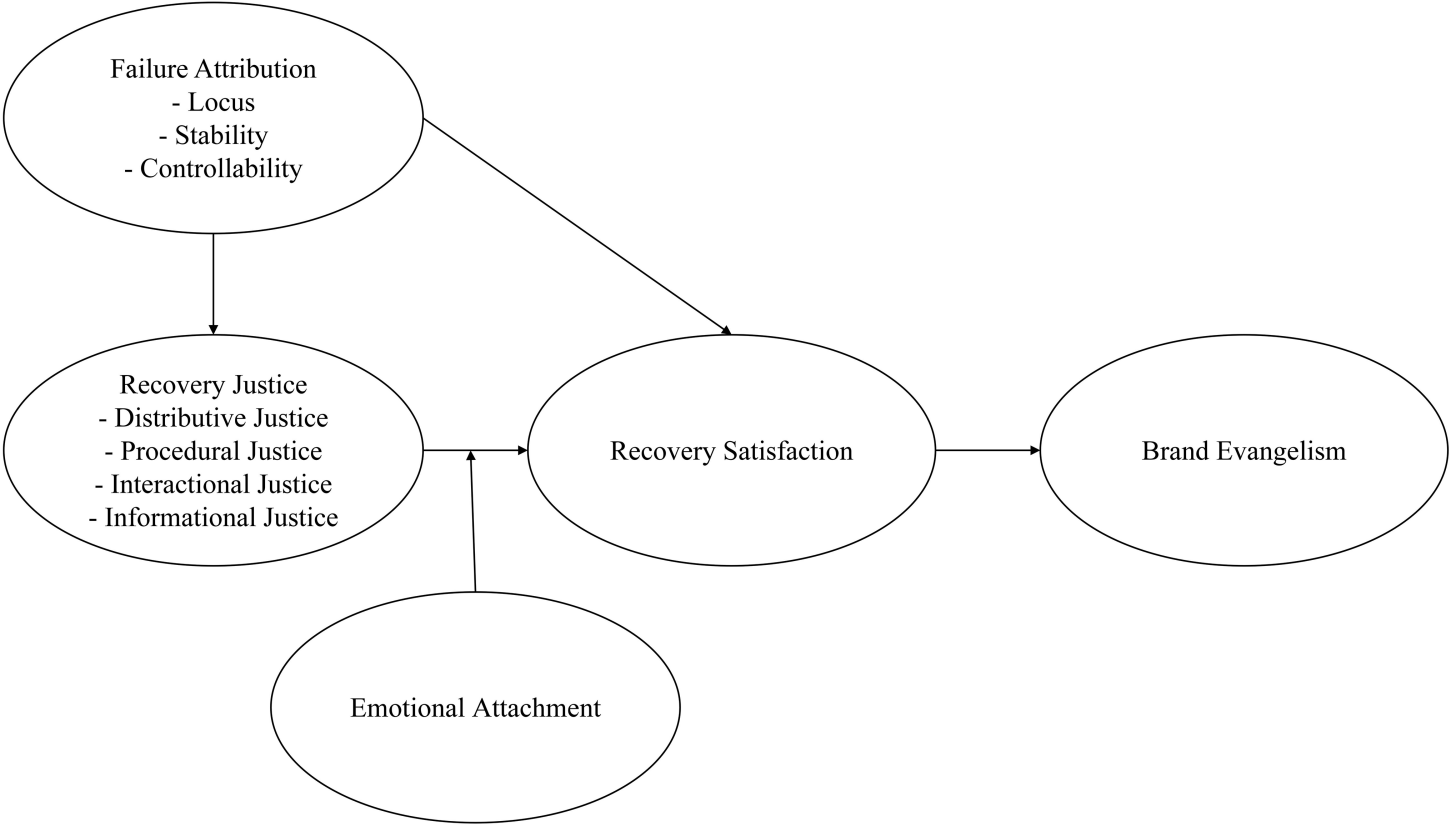
Research model for understanding how failure attribution and recovery justice link to brand evangelism.

Based on Attribution theory, Justice theory, and Attachment theory, we propose a sequential framework as illustrated in Figure 1, starting from customers’ failure attribution and recovery justice, and finally deriving brand evangelism. We combine cognitive and affective factors with behavioral factor in the research model. Failure attribution and recovery justice could be regarded as cognitive factors, while recovery satisfaction as an affective factor, brand evangelism as the behavioral consequence of affective factor. This study hypothesized that failure attribution would have a negative correlation with recovery justice, failure attribution and recovery justice would be significantly associated with recovery satisfaction and subsequent brand evangelism, emotional attachment would moderate the relationship between recovery justice and recovery satisfaction. The reasonableness of this model is obvious. Firstly, customer’s failure attribution reduces his/her recovery justice. Secondly, customer’s recovery satisfaction is driven by his/her failure attribution and recovery justice. The more recovery satisfaction he/she has, the more likely he/she has brand evangelism. Thirdly, the relationship between recovery justice and recovery satisfaction might be moderated by customer’s emotional attachment.

### Failure Attribution and Recovery Justice

Attribution theory has been employed to interpret the causal inference about service failure (O’Neill and Mattila, 2004). When service failure occurs, customers are inclined to search for causes of the problem. Thus, failure attribution is a key factor in explaining customers’ behavioral responses to service failure. A better understanding of failure attribution can help service providers develop successful recovery strategies.

Failure attribution affects cognitive outcomes. Recovery justice is commonly regarded as a cognitive concept (Kima and Baker, 2020). Based on prior studies, this study classified recovery justice into procedural justice, distributive justice, interactional justice and informational justice (Nikbin et al., 2015). In accordance with social exchange theory, people weigh the benefits against the costs of social relationships. They try to maximize benefits and minimize costs, and compare the benefit-and-cost ratio to decide whether it is fair or worthwhile (Homans, 1958). Therefore, customers who view service failures as attributable to the service provider and the cause of service failures as stable and controllable, have a higher probability of perceived injustice. In spite of the importance of failure attribution, few studies have empirically examined its role on recovery justice. Weiner (2006) highlights that attribution dimensions not only influence emotions and behaviors, but are also crucial to social judgments (Weiner, 2006). Schneider and Castillo (2015) have concluded that internal attribution positively impacts justice while external attribution exerts a negative effect on justice (Schneider and Castillo, 2015). According to a survey of employees, Burton et al. (2014) pointed out that internal attribution positively influences interactional justice while external attribution negatively impacts (Burton et al., 2014). Cole (2008) indicated that internal and external attribution affect justice dimensions. Therefore, the following hypotheses are proposed:

*H1*: Locus is negatively associated with justice dimensions.

*H1a*: Locus is negatively associated with procedural justice.

*H1b*: Locus is negatively associated with distributive justice.

*H1c*: Locus is negatively associated with interactional justice.

*H1d*: Locus is negatively associated with informational justice.

*H2*: Stability is negatively associated with justice dimensions.

*H2a*: Stability is negatively associated with procedural justice.

*H2b*: Stability is negatively associated with distributive justice.

*H2c*: Stability is negatively associated with interactional justice.

*H2d*: Stability is negatively associated with informational justice.

*H3*: Controllability is negatively associated with justice dimensions.

*H3a*: Controllability is negatively associated with procedural justice.

*H3b*: Controllability is negatively associated with distributive justice.

*H3c*: Controllability is negatively associated with interactional justice.

*H3d*: Controllability is negatively associated with informational justice.

### Failure Attribution and Recovery Satisfaction

Attribution theory has been applied in many fields (Jiang, 2020). When a service failure occurs, customers make causal attributions to search for the cause of the failure. They seek to comprehend why the event has happened (Nikbin et al., 2012). Failure attribution leads to positive and negative outcomes (Malombe and Choudhury, 2020).

Only a small number of studies have delved into the correlation between failure attribution and recovery satisfaction. Based on prior studies, this study classified failure attribution into locus, stability, and controllability (Moliner-Velázquez et al., 2015). Locus refers to whether customers perceive the cause of service failure to be their fault or the firm’s fault (Moliner-Velázquez et al., 2015; Weitzl et al., 2018; Matikiti et al., 2019). Given that customers are inclined to blame the firm rather than themselves for the cause of service failure, this study focus on external attribution. If there are inconsistencies between recovery efforts and recovery expectations, it will result in customer dissatisfaction (Swanson and Hsu, 2011). So we assume that there is a negative correlation between locus and recovery satisfaction if the cause of failure lies with the firm.

Stability is defined as whether customers consider the cause of service failure as constant. If the cause of service failure is perceived as permanent, customers think that the same service failure will repeatedly occur. It increases customer dissatisfaction. In this situation, customers would likely to claim compensation from the service provider (Hess et al., 2003; Van Vaerenbergh et al., 2014; Moliner-Velázquez et al., 2015; Weitzl et al., 2018; Matikiti et al., 2019; Jiang, 2020). Therefore, this study assumes the presence of a negative correlation between stability and recovery satisfaction, if the cause of failure is permanent.

Controllability is defined as whether customers consider the cause of failure be prevented or controlled by the firm. Customers will express dissatisfaction and tend to blame service providers if they believe that the causes of failure can be controlled by the service provider (Nikbin et al., 2014, 2015; Ngahu, 2019; Jiang, 2020; Malombe and Choudhury, 2020; Gidaković and Čater, 2021). So this study assumes the presence of a negative correlation between controllability and recovery satisfaction, if the cause of failure is controllable. Therefore, the following hypotheses are proposed:

*H4*: Failure attribution is negatively associated with recovery satisfaction.

*H4a*: Locus is negatively associated with recovery satisfaction.

*H4b*: Stability is negatively associated with recovery satisfaction.

*H4c*: Controllability is negatively associated with recovery satisfaction.

### Recovery Justice and Recovery Satisfaction

Justice theory has gained extensive attention in interpreting formation of customer satisfaction. A number of studies have revealed the significant impact of recovery justice on recovery satisfaction in the offline context (Cheung and To, 2016; Balaji et al., 2018), this study investigates if the rule applies to the online context.

Distributive justice is defined as the perceived fairness of the outcomes received by a person (Lin et al., 2011; Nikbin et al., 2012). In connection with service recovery measures, customers judge whether the outcomes provided by service providers are fair (Wu et al., 2020). Rashid and Ahmad (2014) stated that the key to distributive justice is the compensation provided to customers for losses and inconveniences caused by a service failure (Rashid and Ahmad, 2014). Noone (2012) and Bambauer-Sachse and Rabeson (2015) highlighted that compensation effectively alleviates customer dissatisfaction after a service failure (Noone, 2012; Bambauer-Sachse and Rabeson, 2015). Compensation includes a refund, exchange, repair, discount, coupon, etc. Prior studies have pointed out that distributive justice positively affects recovery satisfaction (Wen and Chi, 2013; Tsai et al., 2014; Esen and Sonmezler, 2017; Rashid et al., 2017; Azzahro et al., 2020; Gidaković and Čater, 2021). Therefore, we hypothesize:

*H5a*: Distributive justice is positively associated with recovery satisfaction.

Procedural justice is defined as policies and procedures in dealing with service failure and responding to customer complaints (Nikbin et al., 2012). In other words, it means customer’s perception of the recovery process, recovery policy or rules (Ofori et al., 2015). When service providers admit mistakes, try to correct them, and adapt recovery strategies according to customer needs, customers perceive procedural justice of service recovery. Procedural justice is evaluated according to whether customers are free to express their opinions, transparency in the recovery process, and appropriateness of recovery measures (Tax et al., 1998; Smith et al., 1999; Maxham and Netemeyer, 2003). Previous studies have pointed out that procedural justice positively influences recovery satisfaction (Lii et al., 2012; Wen and Chi, 2013; Esen and Sonmezler, 2017; Rashid et al., 2017; Azzahro et al., 2020; ALhawbani et al., 2021; Badawi et al., 2021; Gidaković and Čater, 2021). Therefore, we hypothesize:

*H5b*: Procedural justice is positively associated with recovery satisfaction.

Interactional justice refers to how fair the customer feels in communicating and coping with service employees after service failure (Kuo and Wu, 2012; Tsai et al., 2014). When service providers demonstrate their politeness, honestness, and empathy in communicating and resolving problems, customers perceive interactional justice of service recovery. Colquitt (2001) found that the expression “I’m sorry” from employees improves recovery satisfaction through interactional justice, because customers use empathy as a criterion for assessing interactional justice (Colquitt, 2001). Interactional justice is also evaluated by credibility, attitude to mistakes, politeness, close attention to solving problems, and a willingness to listen to customer complaints (Smith et al., 1999; Wirtz and Mattila, 2004). Researchers have concluded that interactional justice positively affects recovery satisfaction (Lii et al., 2012; Tsai et al., 2014; Esen and Sonmezler, 2017; Rashid et al., 2017; Azzahro et al., 2020; Olatunde and Nkamnebe, 2021). Therefore, we hypothesize:

*H5c*: Interactional justice is positively associated with recovery satisfaction.

Informational justice focuses on the description of the information provided by service providers about how the procedures were used or how the outcomes were distributed (Colquitt, 2001). Informational justice conveys credibility by reducing secrecy and dishonesty. Customers’ voluntary and favorable evaluation of service recovery and their experiences during service recovery are the leading causes of recovery satisfaction (Bhatti and Khattak, 2015). In terms of informational justice, even when customers experience service failure, they are willing to accept it as fair if they think that the service provider has provided a clear and complete explanation (Bies and Shapiro, 1987). However, informational justice has got little attention in service recovery studies. It is pointed out that customers will perceive more justice if provided with information that helps them make decisions (Gohary et al., 2016b). Few studies have documented that informational justice positively impacts recovery satisfaction in the physical environment (Ofori et al., 2015; Badawi et al., 2021). Amin and Piaralal (2021) suggested that informational justice positively impacts recovery satisfaction in the open and distant learning environment (Amin and Piaralal, 2021). Therefore, we hypothesize:

*H5d*: Informational justice is positively associated with recovery satisfaction.

### Recovery Satisfaction and Brand Evangelism

Service recovery studies have used justice theory as the main framework. According to justice theory, customers with recovery satisfaction are inclined to spread positive word-of-mouth (Moliner-Velázquez et al., 2015). Although brand evangelism originates from psychology, it is regarded as an advanced form of word-of-mouth by marketing scholars. Customers satisfied with service recovery participate in positive word-of-mouth behavior (De Matos and Vargas Rossi, 2008). Therefore, if consumers are satisfied with service recovery, they would like to share their positive experiences with others and become brand evangelists who voluntarily promote the brand. Few studies have demonstrated that recovery satisfaction positively affects brand evangelism (Rashid and Ahmad, 2014; Rashid et al., 2017). Therefore, we hypothesize:

*H6*: Recovery satisfaction is positively associated with brand evangelism.

### The Moderating Role of Emotional Attachment

In line with Consumer–brand relationships theory, consumers establish brand relationships in a way similar to social relationships (Fournier, 1998). Strong emotional associations between consumers and brands induce the development of emotional attachment to the brands (Thomson et al., 2005). In addition, according to Self-expansion theory (Aron and Aron, 1986), consumers are likely to include brands in themselves to build close relationships, which implies the significance of developing emotional attachment between consumers and brands (Loh et al., 2021).

Although the role of emotional attachment as an antecedent or mediator has been well-documented, little research has been done to scrutinize its moderating role. This study is based on “the love is blind” effect, which was suggested by Gregoire and Fisher (2006). Positive emotion towards a service provider significantly influences the impact of recovery efforts. Customers with close connections with service providers do not want to bring the relationships to an end, so they are more likely to forgive service providers for failures (Gregoire and Fisher, 2006). Esen and Sonmezler (2017) declared that emotional attachment moderates the correlation between perceived justice and recovery satisfaction (Esen and Sonmezler, 2017). So we assume that customers with emotional attachment to service providers trade off the negative influences of service failure to maintain close relationships, and respond more favorably to recovery efforts. Therefore, we hypothesize:

*H7*: Emotional attachment moderates the correlation between perceived justice and recovery satisfaction.

*H7a*: Emotional attachment moderates the correlation between procedural justice and recovery satisfaction.

*H7b*: Emotional attachment moderates the correlation between distributive justice and recovery satisfaction.

*H7c*: Emotional attachment moderates the correlation between interactional justice and recovery satisfaction.

*H7d*: Emotional attachment moderates the correlation between informational justice and recovery satisfaction.

## Methodology

### Data Collection

From May 6 to 21, 2021, our research team conducted a 15-day face-to-face questionnaire survey to examine the responses of e-shoppers. Data was collected from e-shoppers who have online service failure and recovery experience in the last year. A pilot study was implemented with 35 qualified undergraduate students to ensure the clarity and validity of the questions. During the pilot study, we saw respondents in person and discussed all questions in detail. Data analysis was performed, and the results of analysis were in accordance with expectations on the whole. Besides, most respondents claimed that the questionnaire was readable and understandable. Combined with statistical analysis and interview feedback, a total of 46 questions were ultimately compiled in the officially released questionnaire, of which 43 were related to the constructs. Specifically, failure attribution (locus, stability, and controllability) and perceived justice (distributive, procedural, interactional, and informational justice) have four questions, respectively. While recovery satisfaction, brand evangelism, and emotional attachment have five questions, respectively. The demographic information of each respondent was gathered and processed as control variables, including gender, age, and monthly income (Gong and Yi, 2019).

In order to reduce the amount of recall bias, we picked out participants on the basis of whether they had encountered at least a service failure and subsequently a service recovery in the e-shopping process over the past year. To be specific, respondents should fulfil two criteria: first, they should have e-shopping service failure experience (e.g., out-of-stock, size mismatch, product defect, exaggerated or false advertising, wrong or delayed delivery, and website error) within 1 year. Second, they should subsequently receive some service recovery measures (e.g., compensation, refund, replacement, and apology) provided by e-shops. A screening question was asked at the start of the survey to determine whether the participants are qualified to answer the questionnaire. The participants without e-shopping service failure and recovery experience or had encountered an e-shopping service failure and recovery over a year ago were not qualified, so they had to be excluded from the data collection. Due to no list of e-shoppers who have service failure and recovery experience, non-probability convenience sampling method was employed. In addition, this method is fast, cost-effective and convenient.

Around 450 questionnaires were distributed, 423 were recollected, and 23 questionnaires with incomplete or suspected unreal answers were excluded. Finally, 400 were effective, representing an 88.9 percent valid response rate. All participants claimed that they had experienced more than one service failure and recovery during e-shopping in the past 1 year. Over half of the participants were female (58.5%), while 41.5% were male. Most respondents were aged 30–39 (34.7%), whereas respondents aged under 29 account for 27% of the total, 24.3% aged 40–49, 14% are older than 50. In addition, 123 (30.8%) of the respondents’ average monthly income is 2,501–5,000 yuan, 90 (22.5%) of them earn 5,001–10,000 yuan, 73 (18.3%) of them earn less than 2,500 yuan, 69 (17.2%) of them earn 10,001–15,000 yuan, while 45 (11.2%) of them earn more than 15,001 yuan.

### Measures

To increase the reliability and validity of data collection, most of the items in the designed questionnaire were derived from previously used questionnaires but have been modified based on the research purpose. We used 5-point Likert scales to measure constructs, which range from “1” denoting complete agreement, and “5” representing complete disagreement.

#### Failure Attribution

Failure attribution is defined as cognitive explanations for service failure (Lee and Cranage, 2018). The scale for failure attribution was adapted from Nikbin et al. (2014). Locus is measured by four items: “I perceive that the problem was caused by me (loc1),” “I perceive that the problem was caused by the e-retailer (loc2),” “I perceive that the problem was caused by an e-shop procedure (loc3),” “I perceive that the problem was caused by an e-shop policy (loc4).” Stability is measured by four items: “The cause of the problem is possible to happen very often (sat1),” “The cause of the problem is possible to occur at a future date (sat2),” “The cause of the problem is possible to be temporary (sta3),” “The cause of the problem is possible to remain unchanged over time (sta4).” Controllability is measured by four items: “The e-retailer could have easily guarded against the cause of the problem (con1),” “The cause of the problem was controllable (con2),” “The cause of the problem was avoidable (con3),” “The cause of the problem was preventable (con4).”

#### Recovery Justice

Distributive justice means the perceived fairness of service recovery’s actual results or consequences (Esen and Sonmezler, 2017). The scale for distributive justice was derived from Esen and Sonmezler (2017), and made up of four items: “I believe the e-retailer was very fair when compensating me for the service failure (dis1),” “In consideration of the trouble caused and the time spent, the compensation I obtained from the e-retailer was suitable (dis2),” “The e-retailer’s efforts sufficed to provide a satisfactory compensation (dis3),” “The e-retailer took adequate compensation measures to address the issue (dis4).”

Procedural justice means the fairness of formal rules and processes that resolve disputes and allocate resources during the service recovery (Esen and Sonmezler, 2017). The scale for procedural justice was derived from Esen and Sonmezler (2017), and made up of four items: “The e-retailer tried to solve the problem promptly (pro1),” “I believe my problem was solved correctly by the e-retailer (pro2),” “I believe the e-retailer has fair policies in tackling issues (pro3),” “I believe that the e-retailer’s complaint handling procedure was adequate (pro4).”

Interactional justice is assessing the degree to which consumers have experienced justice in the interpersonal interactions with employees in the service recovery process (Nikbin et al., 2012). The scale for interactional justice was derived from Gohary et al. (2016a), and composed of four items: “The employees gave a fair amount of concern to my problem (int1),” “The employees listened attentively to my complaint (int2),” “The employees gave me a genuine apology (int3),” “The employees make ample effort to solve my problem (int4).”

Informational justice means the perceived suitability of the information used to explain the cause of the problem during service recovery (Hess et al., 2003). The scale for informational justice was derived from Gohary et al. (2016a), and composed of four items: “The employee explained the procedures thoroughly (inf1),” “The employee’s explanations about the procedures were ample (inf2),” “The employee has been honest in (his/her) conversations with me (inf3),” “The employee delineated the details of the service recovery fully and promptly (inf4).”.

#### Recovery Satisfaction

Recovery satisfaction means the degree of satisfaction with a service provider’s business-specific service recovery attempt (Esen and Sonmezler, 2017). The scale for recovery satisfaction was adapted from Esen and Sonmezler (2017), and composed of four items: “I am satisfied with the way service failure was solved (rec1),” “It seems to me that the e-retailer offered a satisfactory solution to the problem (rec2),” “I have no regrets about choosing this e-shop (rec3),” “Presently, I develop a more positive attitude to this e-shop (rec4),” “I am satisfied with how my problem was handled and solved (rec5).”

#### Brand Evangelism

Brand evangelism is defined as a more proactive and devoted way of broadcasting positive word-of-mouth and fervently attempting to advise others to get engaged with the brand (Becerra and Badrinarayanan, 2013). The scale for brand evangelism was adapted from Becerra and Badrinarayanan (2013), and composed of four items: “Soon, I would probably buy from this e-shop (eva1),” “I spread public praise about this e-shop (eva2),” “I suggest this e-shop to my friends (eva3),” “If my friends were searching goods on the Internet, I would tell them to buy from this e-shop (eva4),” “I would like to tell others that this e-shop is the best in the world (eva5).”

#### Emotional Attachment

Emotional attachment means the extent of an emotion-laden tie between service providers and consumers (Esen and Sonmezler, 2017). The scale for emotional attachment was adapted from Esen and Sonmezler (2017), and composed of five items: “My feelings toward the e-retailer can be characterized by connection (att1),” “My feelings toward the e-retailer can be characterized by passion (att2),” “My feelings toward the e-retailer can be characterized by affection (att3),” “I have no particular feelings about this e-shop (att4),” “My feelings toward the e-retailer can be characterized by delight (att5).”

## Analysis Results and Hypothesis Testing

### Validity and Reliability of Measurements

Cronbach’s alpha was employed to assess the internal consistency of the scales. According to Bagozzi and Yi (1988), reliability scores above 0.60 are adequate (Bagozzi and Yi, 1988). As listed in Table 1, emotional attachment has the highest Cronbach’s Alpha of 0.894, followed by stability 0.861, brand evangelism 0.836, procedural justice 0.829, recovery satisfaction 0.814, distributive justice 0.796, interactional justice 0.791, locus 0.779, controllability 0.728, and informational justice with the lowest Cronbach’s Alpha of 0.717. All items were accepted grounded on Cronbach’s Alpha over 0.70, which indicates satisfactory reliability.

**Table 1:** Results of reliability and validity analysis.

Constructs	Numbers of initial items	Numbers after reliability analysis	Cronbach’*α* value	Numbers after exploratory factor analysis	Numbers after confirmatory factor analysis
Locus	4	4	0.779	4	3
Stability	4	4	0.861	3	3
Controllability	4	4	0.728	4	3
Distributive Justice	4	4	0.796	4	3
Procedural Justice	4	4	0.829	4	3
Interactional Justice	4	4	0.791	3	3
Informational Justice	4	4	0.717	4	3
Recovery Satisfaction	5	5	0.814	5	3
Brand Evangelism	5	5	0.836	5	3
Emotional Attachment	5	5	0.894	5	4

To examine the validity, exploratory factor analysis was performed with exogenous variables and endogenous variables. Based on the analysis results, one item of each locus and interactional justice (loc2 and int1) was excluded. The variance, which can be explained with 10 factors, was 63.8%. Moreover, Kaiser-Meyer-Olkin = 0.819, Bartlett = 7009.731, *df* = 820, *p* = 0.000.

This study utilized confirmatory factor analysis to assess unidimensionality. As shown in Table 2, stability (sat1), controllability (con1), distributive justice (dis2), procedural justice (pro3), informational justice (inf1), and emotional attachment (emo5) were deleted one item. In contrast, recovery satisfaction (resat1 and resat5) and brand evangelism (evan2 and evan3) were deleted two items. The results indicated that the overall fit index reaches a satisfactory level of fitness: *x*^2^ = 588.188 (*p* = 0.00), *df* = 389, GFI = 0.915, AGFI = 0.892, CFI = 0.954, SRMR = 0.048, RMSEA = 0.036.

**Table 2:** Results of confirmatory factor analysis.

Variables	Items	Unstandardized loading	S.E.	*t*-value	Standardized loading	CR	AVE
Locus	loc1 loc3 loc4	1.000 0.880 0.740	– 0.051 0.087	– 17.370 8.524	0.764 0.717 0.648	0.754	0.506
Stability	sta2 sta3 sta4	1.000 0.736 0.982	– 0.063 0.070	– 11.714 14.025	0.799 0.627 0.805	0.790	0.560
Controllability	con2 con3 con4	0.768 0.792 1.000	0.099 0.095 –	7.765 8.332 –	0.694 0.726 0.798	0.784	0.549
Distributive Justice	dis1 dis2 dis4	1.000 0.967 0.723	– 0.075 0.081	– 12.825 8.900	0.777 0.760 0.618	0.764	0.521
Procedural Justice	pro2 pro3 pro4	1.000 0.945 0.917	– 0.060 0.069	– 15.669 13.234	0.824 0.797 0.670	0.809	0.588
Interactional Justice	int2 int3 int4	0.924 1.000 0.973	0.085 – 0.097	10.818 – 10.085	0.694 0.826 0.798	0.818	0.600
Informational Justice	inf2 inf3 inf4	0.745 1.000 0.926	0.054 – 0.062	13.771 – 14.897	0.707 0.852 0.805	0.830	0.625
Recovery Satisfaction	sat2 sat3 sat4	1.000 0.743 0.890	– 0.069 0.080	– 10.757 11.076	0.748 0.652 0.720	0.750	0.501
Brand Evangelism	eva1 eva4 eva5	0.924 1.000 0.930	0.081 – 0.078	11.351 – 11.908	0.726 0.752 0.744	0.785	0.549
Emotional Attachment	emo1 emo2 emo3 emo4	1.000 0.791 0.985 0.971	– 0.054 0.056 0.054	– 14.734 17.485 17.982	0.838 0.697 0.801 0.822	0.870	0.626

Hair et al. (2006) put forward that a proposed model should be evaluated based on convergent validity and discriminant validity. The evaluation of convergent validity should be made by investigating the composite reliabilities and the AVE for each construct (Hair et al., 2006). As listed in Table 2, all the constructs’ composite reliability values surpassed the 0.7 thresholds, and all the values of AVEs exceeded the 0.5 thresholds. Therefore, we can know that the measurement model has an acceptable level of convergent validity, thereby establishing the convergent validity.

Heterotrait Monotrait Ratio (HTMT) method was employed to check the discriminant validity because of its advantage over other methods (Hair et al., 2019). As listed in Table 3, HTMT values of each construct are below the threshold of 0.85, implying that all constructs have sufficient discriminant validity.

**Table 3:** Discriminant validity (HTMT criteria).

Variables	1	2	3	4	5	6	7	8	9	10
Locus (1)	0.711*									
Stability (2)	0.067^**^	0.748^*^								
Controllability (3)	0.275^**^	0.126^**^	0.741^*^							
Distributive Justice (4)	0.384^**^	0.120^**^	0.441^**^	0.722^*^						
Procedural Justice (5)	0.478^**^	0.109^**^	0.364^**^	0.301^**^	0.767^*^					
Interactional Justice (6)	0.419^**^	0.121^**^	0.404^**^	0.354^**^	0.321^**^	0.775^*^				
Informational Justice(7)	0.057^**^	0.102^**^	0.089^**^	0.042^**^	0.065^**^	0.011^**^	0.791^*^			
Recovery Satisfaction(8)	0.073^**^	0.118^**^	0.161^**^	0.098^**^	0.075^**^	0.198^**^	0.056^**^	0.708^*^		
Brand Evangelism (9)	0.127^**^	0.100^**^	0.098^**^	0.068^**^	0.089^**^	0.054^**^	0.107^**^	0.699^**^	0.741^*^	
Emotional Attachment(10)	0.035^**^	0.151^**^	0.140^**^	0.132^**^	0.093^**^	0.112^**^	0.138^**^	0.153^**^	0.104^**^	0.791^*^

* Values on the diagonal are square root of the AVE;

** the off-diagonals are correlations.

### Common Method Bias Testing

Harman’s one-factor test was conducted for assessing the common method bias. We entered all measurement scales into a principal component analysis and interpreted the unrotated factor solution to determine whether the constructs demonstrated common method variance (Podsakoff and Organ, 1986). Results indicated that ten factors emerged with eigenvalues more than 1.0, and the first principal component explained 15.6% of the variance, implying that the possibility of common method bias in this study was low. What’s more, we also employed confirmatory factor analysis to further verify the results. Fit indices of the ten-factor model (*x*^2^/*df* = 1.512, GFI = 0.915, AGFI = 0.892, CFI = 0.954, SRMR = 0.048, and RMSEA = 0.036) were significantly better than the single-factor model (*x*^2^/*df* = 7.869, GFI = 0.604, AGFI = 0.547, CFI = 0.314, SRMR = 0.133, and RMSEA = 0.131), demonstrating that common method bias was not serious in this research.

### Hypothesis Testing

The data was analyzed by structural equation modeling using AMOS 25.0. According to the results of hypothesis testing displayed in Table 4, the overall fit index shows an acceptable level of fitness: *x*^2^ = 550.221 (*p* = 0.00), *df* = 301, GFI = 0.907, AGFI = 0.884, CFI = 0.930, RMR = 0.036, RMSEA = 0.046.

**Table 4:** Analysis results of hypothesis testing.

Hypothesis	Coefficient	*t*-value	Support
H1a: Locus → Distributive Justice	−0.152	−2.538^**^	Yes
H1b: Locus → Procedural Justice	−0.143	−2.943^*^	Yes
H1c: Locus → Interactional Justice	−0.137	−2.423^**^	Yes
H1d: Locus → Informational Justice	−0.015	−0.277	No
H2a: Stability → Distributive Justice	−0.283	−6.641^*^	Yes
H2b: Stability → Procedural Justice	−0.095	−2.440^**^	Yes
H2c: Stability → Interactional Justice	−0.140	−2.733^*^	Yes
H2d: Stability → Informational Justice	−0.086	−1.495	No
H3a: Controllability → Distributive Justice	−0.585	−7.220^*^	Yes
H3b: Controllability → Procedural Justice	−0.826	−7.763^*^	Yes
H3c: Controllability → Interactional Justice	−0.771	−6.889^*^	Yes
H3d: Controllability → Informational Justice	−0.010	−0.113	No
H4a: Locus → Recovery Satisfaction	−0.337	−5.136^*^	Yes
H4b: Stability → Recovery Satisfaction	−0.143	−2.307^**^	Yes
H4c: Controllability → Recovery Satisfaction	−0.492	−2.160^**^	Yes
H5a: Distributive Justice → Recovery Satisfaction	0.273	2.021^**^	Yes
H5b: Procedural Justice → Recovery Satisfaction	0.217	1.967^**^	Yes
H5c: Interactional Justice → Recovery Satisfaction	0.343	2.510^**^	Yes
H5d: Informational Justice → Recovery Satisfaction	0.062	1.342	No
H6: Recovery Satisfaction → Brand Evangelism	0.745	9.799^*^	Yes

Goodness-of-fit statistics *χ*^2^ = 550.221 (*p* = 0.00), *df* = 301, GFI = 0.907, AGFI = 0.884.

CFI = 0.930, RMR = 0.036, RMSEA = 0.046.

* *p* < 0.01;

** *p* < 0.05.

The regression results indicated that failure attribution explained 60.2, 54.7, 37.1, and 15.9% of the total variance in distributive justice, procedural justice, interactional justice and informational justice, respectively. The regression analysis results for failure attribution and perceived justice demonstrated that the variables jointly explained 39.6% of the total variance in recovery satisfaction. In addition, recovery satisfaction explained 60.8% of the total variance in brand evangelism.

Table 4 presents the analysis results of the hypotheses testing. Hypotheses H1a, H1b, H1c, and H1d predicted relationships between locus and recovery justice. After accounting for the control variables, locus displayed a significantly negative influence on distributive justice (*β* = −0.152, *t* = −2.538, *p* < 0.05), procedural justice (*β* = −0.143, *t* = −2.943, *p* < 0.01), interactional justice (*β* = −0.137, *t* = −2.423, *p* < 0.05).That is, H1a, H1b, and H1c were validated. On the contrary, there was no significant correlation between locus and informational justice (*β* = −0.015, *t* = −0.277, ns). Thus, H1d was rejected.

Moreover, hypotheses H2a, H2b, H2c, and H2d predicted relationships between stability and recovery justice. Stability demonstrated a significant negative influence on distributive justice (*β* = −0.283, *t* = −6.641, *p* < 0.01), procedural justice (*β* = −0.095, *t* = −2.440, *p* < 0.05), interactional justice (*β* = −0.140, *t* = −2.733, *p* < 0.01), but no influence on informational justice (*β* = −0.086, *t* = −1.495, ns). These results supported H2a, H2b, H2c but not H2d.

In addition, hypotheses H3a, H3b, H3c, and H3d predicted relationships between controllability and recovery justice. Controllability negatively affected distributive justice (*β* = −0.585, *t* = −7.220, *p* < 0.01), procedural justice (*β* = −0.826, *t* = −7.763, *p* < 0.01), interactional justice (*β* = −0.771, *t* = −6.889, *p* < 0.01), thus supporting H3a, H3b, and H3c, respectively. Nonetheless, controllability did not have a significant impact on informational justice (*β* = −0.010, *t* = −0.113, ns). H3d was not supported.

Furthermore, hypotheses H4a, H4b, and H4c predicted relationships between failure attribution and recovery satisfaction. Locus (*β* = −0.337, *t* = −5.136, *p* < 0.01), stability (*β* = −0.143, *t* = −2.307, *p* < 0.05), controllability (*β* = −0.492, *t* = −2.160, *p* < 0.05) were significantly related to recovery satisfaction in a negative way. Thus, H4a, H4b, and H4c were statistically supported as hypothesized.

Besides, hypotheses H5a, H5b, H5c, and H5d predicted relationships between recovery justice and recovery satisfaction. Distributive justice (*β* = 0.273, *t* = 2.021, *p* < 0.05), procedural justice (*β* = 0.217, *t* = 1.967, *p* < 0.05), interactional justice (*β* = 0.343, *t* = 2.510, *p* < 0.05) exerted significant and positive relationships with recovery satisfaction. But the influence of informational justice on recovery satisfaction was not significant (*β* = 0.062, *t* = 1.342, ns). These results supported H5a, H5b, H5c but not H5d.

Also, the hypothesized relationship between recovery satisfaction and brand evangelism was significant (*β* = 0.745, *t* = 9.799, *p* < 0.01), thereby supporting H6.

### Moderating Effect Testing

In the second phase, we conducted a multiple-group analysis to examine the moderating role of emotional attachment in the correlation between justice dimensions and recovery satisfaction (H7a, H7b, H7c, and H7d). To conduct the multiple-group analysis, we created two subsamples—high emotional and low emotional attachment- based on the emotional attachment scale by Esen and Sonmezler (2017). The total sample was segmented into two data sets on the basis of the median value. The low emotional attachment group had 214 respondents, while the high emotional attachment group had 186 respondents.

Table 5 presents the analysis results of the moderating role of emotional attachment. It is thought that if the constrained model’s variation of the difference of the chi-square values is statistically significant to a higher degree than the chi-square criteria threshold, the hypothesis is supported (Stone and Hollenbeck, 1989). When analyzing the impact of distributive justice on recovery satisfaction, the analysis result displayed that the constrained model was *χ*^2^ = 931.809 (*df* = 603), while the free model was *χ*^2^ = 927.214 (*df* = 602). The constrained model’s variation of the difference of the chi-square values with one degree of freedom Δ*χ*^2^ (1) was 4.594 (*p* = 0.032). The result was deemed statistically significant, thereby supporting H7a.

**Table 5:** Analysis results of the moderating role of emotional attachment.

Hypothesis	Δ*df*	The chi-square difference
H7a: Distributive Justice → Recovery Satisfaction	Δ*df* = 1	Δ*χ*^2^ = 4.594 (*p* = 0.032)
H7b: Procedural Justice → Recovery Satisfaction	Δ*df* = 1	Δ*χ*^2^ = 8.731 (*p* = 0.003)
H7c: Interactional Justice → Recovery Satisfaction	Δ*df* = 1	Δ*χ*^2^ = 1.065 (*p* = 0.302)

Concerning the impact of procedural justice on recovery satisfaction, according to the result of examining the difference of the chi-square values between the free and constrained model, the constrained model was *χ*^2^ = 935.946 (*df* = 603). In contrast, the free model was *χ*^2^ = 927.214 (*df* = 602). The constrained model’s variation of the difference of the chi-square values with one degree of freedom Δ*χ*^2^(1) was 8.731 (*p* = 0.003). The result was considered to be statistically significant, thereby supporting H7b.

As for the impact of interactional justice on recovery satisfaction, the analysis result revealed that the constrained model was *χ*^2^ = 928.279 (*df* = 603), whereas the free model was *χ*^2^ = 7927.214 (*df* = 602). The Chi-square difference with one degree of freedom between the free and constrained model was statistically insignificant (Δ*χ*^2^(1) = 1.065, *p* = 0.302), meaning that the two groups have similar path coefficients over the conceptual model. It provided evidence for no moderating effect of emotional attachment across the two groups, leading to the rejection of H7c.

Based on the structural model results, informational justice was not related to recovery satisfaction; therefore, the moderating effect of emotional attachment in the correlation between informational justice and recovery satisfaction was not significant. That is, H7d was not supported.

## Discussion

### Findings

To test how failure attribution and recovery justice affect brand evangelism from the perspective of e-shoppers, we utilized the Attribution theory, Justice theory and Attachment theory to develop a conceptual frame in which failure attribution (namely, locus, stability, and controllability) and recovery justice (namely, distributive, procedural, interactional, and informational justice) influence brand evangelism through recovery satisfaction with emotional attachment as a moderator. Results show that three attribution dimensions (locus, stability, and controllability) negatively affect recovery satisfaction, while three justice dimensions (distributive, procedural, and interactional justice) positively affect recovery satisfaction, which is further positively correlated with brand evangelism. As for the relationship between two antecedents, three attribution dimensions (locus, stability, and controllability) negatively affect three justice dimensions (distributive, procedural, and interactional justice). In addition, emotional attachment moderates the relationship between distributive justice-recovery satisfaction and procedural justice-recovery satisfaction. To sum up, research results firmly support the assertion that failure attribution and recovery justice promote brand evangelism by improving recovery satisfaction.

### Implications for Theory

This research makes contributions to previous literature in three aspects. First, prior research of service failure and recovery has only considered two attribution dimensions (namely, stability, and controllability) and three justice dimensions (namely, distributive, procedural, and interactional). Little recent research has empirically examined the influence of failure attribution by including locus as an independent variable (Oflac et al., 2021), and only a minority of studies have empirically investigated the effect of informational justice as an independent variable (Nikbin et al., 2015; Ngahu et al., 2016). Furthermore, empirical studies exploring the impact of failure attribution on recovery justice have rarely been conducted (Burton et al., 2014; Schneider and Castillo, 2015). Our study has made a contribution to service recovery literature by empirically verifying a research model including the third dimension of attribution (locus) and fourth dimension of justice (informational justice), as well as exploring how failure attribution influences recovery justice to demystify the process.

Second, although there is some theoretical research on brand evangelism, empirical studies of brand evangelism are still inadequate (Hsu, 2019; Kang et al., 2020). In particular, brand evangelism is still unexplored in the context of service recovery. Our study has contributed to brand evangelism literature by exploring the ways in which brand evangelism is stimulated or deterred in the process of online service recovery. This study enhances our comprehension of brand evangelism by ascertaining that failure attribution and recovery justice influence brand evangelism by inducing recovery satisfaction.

Third, although many scholars have highlighted the effect of emotional attachment as an independent variable (Wen and Chi, 2013; Nguyen and Minh, 2018) or mediator (Río-Lanza et al., 2009; Vázquez-Casielles et al., 2012), little attention has been paid to the moderating role of emotional attachment (Esen and Sonmezler, 2017). Our study has made a contribution to service recovery literature by considering emotional attachment moderating the relationship between recovery justice and recovery satisfaction, thereby emphasizing the pivotal role of emotional attachment. We offer a precise mechanism of how recovery justice improves customers’ recovery satisfaction with the moderating effect of emotional attachment.

### Implications for Practice

Our study gives some important suggestions for practitioners in implementing effective service recovery in the e-retailing industry. The research findings could be served as a guide for e-retailers to comprehend e-shoppers’ behavior, enhance customer satisfaction and stimulate brand evangelism.

First, failure attribution (locus, stability, and controllability) was found to have a negative effect on recovery justice (distributive, procedural, and interactional justice). In other words, e-shoppers’ recovery justice perceptions are influenced by the attributions they make for the service failures. Specifically, locus was turned out to negatively affect distributive, procedural and interactional justice. Hence, it is of great importance for e-retailers to clearly comprehend the locus of failure from e-shoppers’ perspective. If e-shoppers believe that the problem was caused by e-retailers, their perceptions of recovery justice decrease. Therefore, e-retailers should also take locus of attribution into consideration of service recovery management. When service failure occurs, the first step should be failure detection to find out the root cause of the problems (Zhu and Zolkiewski, 2015). By doing so, e-retailers can detect the shortcomings in business operations and prioritize the works that are in need of improvement, so that it will be possible to monitor and control future service failures (Oflac et al., 2021). In addition, stability was proved to negatively impact distributive, procedural and interactional justice. It means that e-shoppers will be more likely to perceive justice if they think the cause of a service failure as unstable. Therefore, e-retailers should emphasize that service failure is a temporary phenomenon, and develop effective strategies for prevention of recurrence of service failure. Moreover, controllability was concluded to negatively influence distributive, procedural and interactional justice, meaning that it is crucial for e-retailers to avoid service failure controlled by e-shops to guarantee justice to customers. If a customer perceives service failure as controllable, the customer’s image of the e-shop is likely to deteriorate. So it is necessary to emphasize that service failure was not controllable by e-retailer. To provide high quality service, e-retailers should identify the types of service failure that can be caused by controllable and stable causes, and prepare countermeasures thoroughly in advance.

Second, failure attribution (locus of causality, stability, and controllability) and recovery justice (distributive, procedural, and interactional justice) were demonstrated to influence recovery satisfaction, which in turn impacts brand evangelism. More specifically, failure attribution (locus, stability, and controllability) was discovered to have a negative impact on recovery satisfaction, while recovery justice (distributive, procedural, and interactional justice) was proved to positively impact recovery satisfaction. It means that if e-shoppers do not place blame on e-retailers and consider the cause of service failure as unstable and uncontrollable, or if they consider service recovery efforts as fair, they will be likely to have more recovery satisfaction. Therefore, it is crucial that e-retailers should know the likelihood of service failure in advance and provide strategies to prevent controllable and stable service failure (generated by their own fault) occurs. After a service failure, e-retailers should mitigate the negative impact of service failure by providing appropriate complaint-handling efforts to improve customers’ recovery satisfaction (Mazhar et al., 2022). E-retailers should hire kind and polite employees, and also give training and guidance continuously and systematically to ensure service standards during service recovery operations. In addition, it is also essential to compensate customers for the loss or inconvenience caused by a service failure. Compensation is an effective way to guarantee distributive justice and mitigate customer dissatisfaction after a service failure, no matter it is provided in monetary or non-monetary form. Also, procedures or policies of service failure are important in the guarantee of procedural justice. If e-retailers perform effective recovery measures, customer satisfaction will be improved. Furthermore, recovery satisfaction was discovered to positively affect brand evangelism. In other words, recovery satisfaction is a key determinant of brand evangelism. Customers who experience high recovery satisfaction after service failure will show higher brand evangelism than customers who experience low recovery satisfaction. This conclusion aligns with the studies of Rashid and Ahmad (2014) and Rashid et al. (2017). E-retailers should realize that recovery satisfaction could alleviate and balance customers’ dissatisfaction with the service failure (Magnini et al., 2007). Consumers tend to behave positively toward the brands they are satisfied with, while they tend to showcase anti-brand behavior toward the brands they are dissatisfied with. Therefore, even after service failures, there is a high probability to change service failures into experience-rewriting instruments. E-retailers with appropriate failure attribution and recovery justice management will be able to turn the dissatisfied e-shoppers into satisfied ones and even brand evangelists.

Third, the significant moderating effects of the emotional attachment on the correlations between distributive and procedural justice on recovery satisfaction imply that the higher the emotional attachment, the stronger the positive correlations between distributive and procedural justice on recovery satisfaction. If the emotional attachment is low, it will be challenging for e-retailers to earn a high level of recovery satisfaction even with service recovery efforts (Wei, 2021). Therefore, e-retailers should prioritize increasing customers’ emotional attachment. If customers develop emotional attachments to the e-shop, they are inclined to forgive service failure and respond more favorably to e-retailers’ recovery efforts. A strong emotional attachment serves as a protecting net for e-shops when a service fails (Torres et al., 2020). Hence, investment in building emotional attachment with customers is a sensible strategy from service recovery perspective.

## Limitations and Future Research

Despite the interesting results yielded by the research, limitations were also encountered. Firstly, since this study collected data from one service sector (e-retailing industry), some problems may be entailed with the generalization of research findings. Future research should expand to other industries. Secondly, it was challenging to obtain generalized results suitable for a standardized situation by conducting a questionnaire survey. The major event method or scenario method should be used in future research to overcome the inherent deficiencies of questionnaire survey. Finally, this study may be meaningful because informational justice was added to justice dimensions. But the impact of failure attribution on informational justice and the influence of informational justice on recovery satisfaction were not significant in this study. It might be because of this research’s setting. In the e-retailing sector, service failures are so common that explanations may not work (Wang and Mattila, 2011). Therefore, it will be necessary to retest whether the same results are obtained in other sectors.

## Conclusion

The purpose of the study was to determine whether failure attribution and recovery justice are related with brand evangelism in the context of online service recovery and whether emotional attachment moderates the effects. The results suggest that three attribution dimensions (locus, stability, and controllability) negatively affect recovery satisfaction, while three justice dimensions (distributive, procedural, and interactional justice) positively influence recovery satisfaction, which in turn positively impact brand evangelism. As for the correlation between two antecedents, three attribution dimensions (locus, stability, and controllability) negatively affect three justice dimensions (distributive, procedural, and interactional justice). Moreover, emotional attachment moderates the correlation between recovery justice and recovery satisfaction in terms of distributive and procedural justice. Research findings provide empirical evidence that both failure attribution and recovery justice are vital to encourage brand evangelism. This study builds a more comprehensive framework than previous studies by incorporating the third dimension of failure attribution (locus) and the fourth dimension of recovery justice (informational justice), and offers insights into the impact of failure attribution on recovery justice. Moreover, this study expands brand evangelism to service recovery context by examining how brand evangelism is stimulated among e-shoppers and advances our comprehension of brand evangelism by finding the way in which failure attribution and recovery justice contribute to brand evangelism *via* recovery satisfaction. Furthermore, the research finding concerning the moderating effect of emotional attachment bridges a gap in the existing literature which focuses on the effect of emotional attachment as an antecedent or mediator. This study offers valuable guidance for those e-retailers wishing to develop successful strategies for encouraging brand evangelism from service recovery.

## Data Availability Statement

The raw data supporting the conclusions of this article will be made available by the authors, without undue reservation.

## Author Contributions

TZ contributed to the conceptualization, funding acquisition, and investigation. SP contributed to the conceptualization, formal analysis, and methodology. All authors contributed to the article and approved the submitted version.

## Funding

This study was supported by the Humanities and Social Sciences Research Foundation of the Chinese Ministry of Education under grant 21YJCZH252, Philosophical and Social Science Key Foundation of Anhui Province under grant AHSKQ2021D17, the Humanity and Social Science Major Foundation of Education Committee of Anhui Province under grant SK2020A0170, and the Natural Science Foundation of Anhui Province under grant 1908085QG301.

## Conflict of Interest

The authors declare that the research was conducted in the absence of any commercial or financial relationships that could be construed as a potential conflict of interest.

## Publisher’s Note

All claims expressed in this article are solely those of the authors and do not necessarily represent those of their affiliated organizations, or those of the publisher, the editors and the reviewers. Any product that may be evaluated in this article, or claim that may be made by its manufacturer, is not guaranteed or endorsed by the publisher.
